# Optimizing Alkyl Side Chains in Difluorobenzene–Rhodanine Small-Molecule Acceptors for Organic Solar Cells

**DOI:** 10.3390/ma17081875

**Published:** 2024-04-18

**Authors:** Jongchan Choi, Chang Eun Song, Eunhee Lim

**Affiliations:** 1 Department of Chemistry, Kyonggi University, Suwon 16227, Republic of Korea; 2 Korea Research Institute of Chemical Technology, Daejeon 34114, Republic of Korea; songce@krict.re.kr; 3 Department of Applied Chemistry, University of Seoul, Seoul 02504, Republic of Korea

**Keywords:** solar cells, photovoltaic, nonfullerene, acceptor, organic solar cell, rhodanine

## Abstract

A series of small molecules, **T-2FB-T-ORH**, **T-2FB-T-BORH**, and **T-2FB-T-HDRH**, were synthesized to have a thiophene-flanked difluorobenzene (T-2FB-T) core and alkyl-substituted rhodanine (RH) end groups for their use as nonfullerene acceptors (NFAs) in organic solar cells (OSCs). Octyl, 2-butyloctyl (BO), and 2-hexyldecyl (HD) alkyl side chains were introduced into RHs to control the material’s physical properties based on the length and size of the alkyl chains. The optical properties of the three NFAs were found to be almost the same, irrespective of the alkyl chain length, whereas the molecular crystallinity and material solubility significantly differed depending on the alkyl side chains. Owing to the sufficient solubility of **T-2FB-T-HDRH**, OSCs based on PTB7-Th and **T-2FB-T-HDRH** were fabricated. A power conversion efficiency of up to 4.49% was obtained by solvent vapor annealing (SVA). The AFM study revealed that improved charge mobility and a smooth and homogeneous film morphology without excessive aggregation could be obtained in the SVA-treated film.

## 1. Introduction

Solution-processed organic solar cells (OSCs) are attracting significant attention owing to their numerous advantages, including low cost, light weight, and flexibility [1,2,3]. Fullerene acceptors, such as PC_61_BM, have been used owing to their high electron mobility, suitable energy levels, and isotropic charge transfer properties. However, fullerene acceptors suffer from drawbacks such as weak UV–vis absorption, challenging purification processes, high production costs, and limited molecular structure modification capabilities, resulting in difficulties in achieving high device efficiency [4,5]. Therefore, nonfullerene-based acceptors have emerged as promising alternatives because of their strong and broad UV–vis absorption, facile purification processes, cost-effectiveness, and fine-tuning of material properties [6,7]. By blending nonfullerene acceptors (NFAs) with suitable polymer donors, OSCs achieved power conversion efficiencies (PCEs) exceeding 18% [8,9,10,11].

NFAs typically comprise an electron-donating donor (D) group and an electron-withdrawing acceptor (A) group. Common donating groups include electron-rich fluorene [12,13], indacenodithiophene (IDT) [14,15], indacenodithieno[3,2-*b*]thiophene (IDTT) [16,17], benzo[1,2-*b*:4,5-*b*′]dithiophene [18], and dithienothiophen[3.2-b]-pyrrolobenzothiadiazole (TPBT) [19,20]. Electron accepting groups mainly include 2-(2,3-dihydro-3-oxo-1*H*-inden-1-ylidene)-propanedinitrile (INCN), 2-(3-oxo-2,3-dihydroinden-1-ylidene)malononitrile (DCIN), rhodanine (RH), barbituric acid (BAR), and (2-(6-oxo-5,6-dihydro-4H-cyclopenta[*c*]thiophen-4-lidene)malononitrile) (CPTCN) [21,22,23,24]. 

Recently, NFAs have been synthesized in a rod-shaped A–D–A structure. This structure offers advantages such as favored charge separation at the interface between donor and acceptor molecules, easy control of UV–vis absorption and energy levels, and optimal intermolecular aggregation in the solid state [25]. For instance, Peng et al. reported two Y-type NFAs named BTP-Cy-4F and BTP-Cy-4Cl with an A–D–A structure, achieving a high PCE of 19.36%. The fused ring core was designed to have outer branched side chains and inner cyclohexane-based side chains, along with fluorinated or chlorinated DCIN end groups [26]. 

The physical properties of the acceptors vary significantly depending on the side chain introduced. The size and shape of the side chains directly influence crucial factors for OSC fabrication such as material solubility, crystallinity, and charge mobility [27,28,29]. Typically, the molecular core of recently reported high-efficiency acceptors consists of a fused multicyclic ring, into which the long alkyl side chains are introduced to improve material solubility. However, excessively long alkyl groups can enhance material solubility but concurrently diminish molecular crystallinity and charge mobility, potentially resulting in decreased device efficiency. Conversely, if the introduced alkyl side chain is not long enough, it is difficult to provide solubility and film morphology suitable for solution processing because of excessive molecular aggregation, consequently reducing the short-circuit current (*J*_SC_) of the devices. Therefore, it is essential to optimize the length and shape of the side chains to select the side chains for a given molecular backbone to develop an NFA. For instance, Sun et al. reported three acceptors using CPTCN as the end group and varying the side chain of the IDTT core to achieve a PCE of 13.7% [30]. Similarly, a study by Li et al. achieved a PCE of 15.32% by modifying the size of branched alkyl side chains on a BTP core [31]. Our group also reported small-molecule NFAs based on bithiophene and alkylrhodanine as the core and end groups, respectively. We found that the introduction of asymmetric alkyl side chains in the RH end groups not only increased the PCEs of the devices but also enabled nonhalogenated solvent processing [32]. 

In this study, three small A–D–A-type molecules, **T-2FB-T-ORH**, **T-2FB-T-BORH**, and **T-2FB-T-HDRH**, were synthesized, with electron-rich thiophene-flanked difluorobenzene (T-2FB-T) at the core and electron-withdrawing RH groups at both ends. They were designed to have different alkyl (octyl, 2-butyloctyl (BO), and 2-hexyldecyl (HD)) groups, and their physical properties and device performances were compared.

## 2. Experimental Section 

### 2.1. Materials 

Tris(dibenzylideneacetone)dipalladium(0) (Pd_2_(dba)_3_), 2-(tributylstannyl)thiophene, *n*-butyllithium *(n*-BuLi, 1.6 M solution in hexanes), 4-formylmorpholine, triethylamine (TEA), *n*-octylamine, piperidine, and anhydrous solvents such as tetrahydrofuran (THF), toluene, and methanol were purchased from Aldrich (Anseong-si, Gyeonggi-do, Republic of Korea). 1,4-Dibromo-2,5-difluorobenzene, tri(*o*-tolyl)phosphine (P(*o*-tol)_2_), phthalimide, triphenylphosphine (PPh_3_), 2-butyl-*n*-octanol, 2-hexyl-*n*-decanol, hydrazine monohydrate, and bis(carboxymethyl)trithiocarbonate, 1,2-dimethoxyethane (DME) were purchased from Tokyo Chemical Industry (Tokyo, Japan). Diisopropyl azodicarboxylate (DIAD) was purchased from Alfa Aesar (Seoul, Republic of Korea). All syntheses were conducted under a nitrogen atmosphere using anhydrous solvents. *N*-(2-Butyloctyl)phthalimide (BOphth), 2-butyloctylamine (BONH_2_), 3-Octylrhodanine (**ORH**), and 3-(2-butyloctyl)rhodanine (**BORH**) were synthesized according to previous reports [23,33,34,35].

### 2.2. Synthesis

#### 2.2.1. *N*-(2-Hexyldecyl)phthalimide (**HDphth**) [33]

This compound was synthesized in the same manner as **BOphth** according to the previous report [33] using phthalimide (3.6 g, 24 mmol), PPh_3_ (7.8 g, 30 mmol), 2-hexyl-*n*-decanol (6.0 g, 25 mmol), and DIAD (6.0 g, 30 mmol) to produce a colorless oil. Yield: 7.8 g (85%). ^1^H NMR (400 MHz, CDCl_3_): *δ* 7.88–7.80 (*m*, 2H), 7.73–7.65 (*m*, 2H), 3.57 (*d*, *J* = 7.2 Hz, 2H), 1.94–1.84 (*m*, 1H), 1.45–1.17 (*m*, 25H), and 0.90–0.81 (*m*, 6H).

#### 2.2.2. 2-Hexyldecylamine (**HDNH_2_**) [34]

This compound was synthesized in the same manner as **BONH_2_**, according to the previous report [34], using **HDphth** (7.8 g, 21 mmol) and hydrazine monohydrate (3.1 g, 63 mmol) to produce a yellow oil. The compound was used without further purification. Yield: 4.9 g (96%). ^1^H NMR (400 MHz, CDCl_3_): *δ* 2.60 (*d*, *J* = 4.8 Hz, 2H), 1.32–1.24 (*m*, 25H), 1.21 (*s*, 2H), and 0.88 (*m*, 6H).

#### 2.2.3. 3-(2-Hexyldecyl)rhodanine (**HDRH**) [23,36]

This compound was synthesized in the same manner as **BORH,** according to the previous reports [23,36], using bis(carboxymethyl)trithiocarbonate (4.5 g, 20 mmol), DME (40 mL), TEA (2.0 g, 20 mmol), and **HDNH_2_** (4.8 g, 20 mmol). Column chromatography (DCM:hexane = 1:1) was conducted, resulting in a yellow oil. Yield: 6.2 g (87%). ^1^H NMR (400 MHz, CDCl_3_): *δ* 3.97 (*s*, 2H), 3.89 (*d*, *J* = 7.4 Hz, 2H), 2.07–1.99 (*m*, 1H), 1.35–1.20 (*m*, 24H), and 0.91–0.85 (*m*, 6H). 

#### 2.2.4. 2,2′-(2,5-Difluoro-1,4-phenylene)dithiophene (**T-2FB-T**) [36] 

1,4-Dibromo-2,5-difluorobenzene (0.80 g, 2.9 mmol), Pd_2_(dba)_3_ (0.16 g, 0.17 mmol), P(*o*-tol)_2_ (0.09 g, 0.32 mmol), and 2-(tributylstannyl)thiophene (2.4 g, 6.6 mmol) were dissolved in toluene (35 mL) and refluxed overnight at 90 °C. The mixture was then extracted with chloroform and water, followed by the removal of water using MgSO_4_. The product was further purified by column chromatography (DCM:hexane = 1:2) to produce a white solid. Yield: 0.50 g (61%). ^1^H NMR (400 MHz, CDCl_3_): *δ* (ppm) 7.52 (*d*, *J* = 3.7 Hz, 2H), 7.44–7.38 (m, 4H), and 7.14 (*dd*, *J* = 5.1 and 3.7 Hz, 2H). 

#### 2.2.5. 5,5′-(2,5-Difluoro-1,4-phenylene)bis(thiophene-2-carbaldehyde) (**T-2FB-T-CHO**) [37]

**T-2FB-T** (0.5 g, 1.8 mmol) was completely dissolved in distilled THF (30 mL). The temperature was lowered to –78 °C, and *n*-BuLi in hexane (1.6 M, 2.6 mL, 4.1 mmol) was added dropwise. After stirring for 2 h, 4-formylmorpholine (0.47 g, 4.1 mmol) was added dropwise, and stirring continued for 1 h. Stirring was then carried out at room temperature for 2 h, followed by neutralization with 1 M HCl. After removing all the solvent, a yellow solid was obtained through recrystallization with methanol. Yield: 0.51 g (85%). ^1^H NMR (400 MHz, CDCl_3_): *δ* 9.96 (*s*, 2H), 7.80 (*d*, *J* = 3.8 Hz, 2H), 7.63 (*d*, *J* = 3.8 Hz, 2H), and 7.52 (*t*, *J* = 8.7 Hz, 2H). 

#### 2.2.6. Synthesis of **T-2FB-T-ORH** [32,38]

**T-2FB-T-CHO** (0.21 g, 0.62 mmol) was dissolved in chloroform (100 mL). Three drops of piperidine were added, followed by the addition of **ORH** (1.2 g, 5.0 mmol). The mixture was refluxed for 24 h. After removing all the solvent, recrystallization was performed using chloroform and methanol to obtain a red solid. Yield: 0.31 g (62%).

#### 2.2.7. Synthesis of **T-2FB-T-BORH**

The synthesis of this compound followed the same procedure as **T-2FB-T-ORH**, utilizing **T-2FB-T-CHO** (0.20 g, 0.59 mmol) and **BORH** (1.4 g, 4.8 mmol) to produce a red solid. Yield: 0.30 g (56%). ^1^H NMR (400 MHz, CDCl_3_): *δ* (ppm) 3.97 (s, 2H), 3.89 (*d*, *J* = 7.4 Hz, 2H), 2.07–1.99 (*m*, 1H), 1.35–1.20 (*m*, 24H), and 0.91–0.85 (*m*, 6H).

#### 2.2.8. Synthesis of **T-2FB-T-HDRH**

The synthesis of this compound followed the same procedure as **T-2FB-T-ORH**, utilizing **T-2FB-T-CHO** (0.20 g, 0.59 mmol) and **HDRH** (1.7 g, 4.8 mmol) to produce a red solid. Yield: 0.42 g (69%). ^1^H NMR (400 MHz, CDCl_3_): *δ* (ppm) 3.97 (*s*, 2H), 3.89 (*d*, *J* = 7.4 Hz, 2H), 2.07–1.99 (*m*, 1H), 1.35–1.20 (*m*, 24H), and 0.91–0.85 (*m*, 6H). Anal. Calc. for C_54_H_74_F_2_N_2_O_2_S_6_: C, 63.99; H, 7.36; N, 2.76; and S, 18.98. Found: C, 63.98; H, 7.13; N, 2.65; and S, 18.61.

### 2.3. Measurement

The nuclear magnetic resonance (NMR) spectra of all the intermediates and the final compounds were recorded on a Bruker Avance II 400 spectrometer (Billerica, MA, USA), and the elemental analysis (EA) was performed using a Vario-EL III instrument at the Korea Basic Science Institute (Pusan, Republic of Korea). Thermogravimetric analysis (TGA) and differential scanning calorimetry (DSC) were conducted using a Mettler-Toledo Inc. instrument (Columbus, OH, USA) under a nitrogen atmosphere with heating and cooling rates of 10 °C/min. UV–vis spectra were obtained using a Mega-U600 spectrometer (Auckland, New Zealand), and UV–vis films were prepared by spin-coating the solid materials dissolved in chloroform onto glass substrates at 4000 rpm. Cyclic voltammetry (CV) measurements were carried out using a BAS 100B instrument (Bioanalytical Systems, IN, USA) with a nonaqueous reference electrode (0.1 M Ag/Ag+ in acetonitrile) and platinum working and counter electrodes. For the measurement of the oxidation–reduction potential of the final compounds, 0.1 M Tetra-*n*-butylammonium hexafluorophosphate in acetonitrile was used as the electrolyte. The small molecules dissolved in the chloroform solvent were drop-cast onto a platinum electrode, and measurements were calibrated using a ferrocenium/ferrocene value of –4.8 eV as an external reference. The highest occupied molecular orbital (HOMO) and lowest unoccupied molecular orbital (LUMO) energy levels were estimated according to the empirical relationships *E*_HOMO_ = −(*E*_onset,ox_ − *E*_1/2,ferrocene_ + 4.8) eV and *E*_LUMO_ = −(*E*_onset,red_ − *E*_1/2,ferrocene_ + 4.8) eV, where *E*_onset,ox_ and *E*_onset,red_ are the onset potentials of oxidation and reduction, respectively, assuming that the energy level of ferrocene is 4.8 eV below the vacuum level. Atomic force microscopy (AFM) (Salt Lake City, UT, USA) images were taken using a scanning probe microscope (XE-100).

### 2.4. Fabrication of OSC Devices

The OSCs were fabricated in an inverted architecture of ITO/ZnO nanoparticles. (NPs)/polyethylenimine (PEIE, 80% ethoxylated)/PTB7-Th:acceptor/MoO*_x_*/Ag. ZnO NPs were spin-coated in sol–gel form and annealed at 200 °C for 60 min. The photoactive layer was prepared by blending the donor material PTB7-Th with the small-molecule acceptor in chloroform solvent, spin-coated at 4000 rpm using a 0.45 μm PTFE membrane syringe filter, and subjected to solvent vapor annealing (SVA) for 15 s or thermal annealing (TA) at 90 °C for 10 min under the chloroform condition. After each treatment, MoO*_x_* and Ag were vacuum-deposited. The active area was 3 × 3 mm^2^. The current density−voltage (*J*−*V*) characteristics were measured using a Keithley 236 (Solon, OH, USA) under AM 1.5 G illumination (10 mW/cm^2^). External quantum efficiency (EQE) was measured using a reflective microscope, focusing the light output from a monochromator and optical chopper onto a 100 W halogen lamp. 

## 3. Results and Discussion

### 3.1. Synthesis and Thermal Properties

**Scheme 1:**
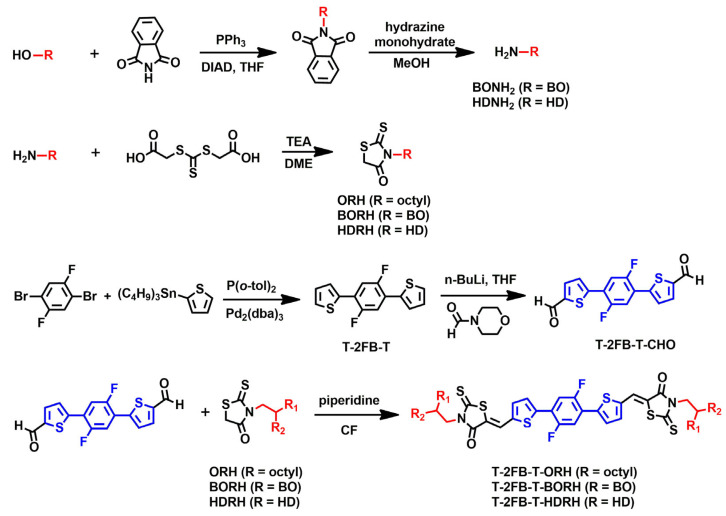
Synthetic procedures for T-2FB-T-ORH, T-2FB-T-BORH, and T-2FB-T-HDRH.

A series of small molecules were designed to have a T-2FB-T core and alkyl-substituted RH end groups for use as acceptors in OSCs. They had the same molecular backbone, but three different alkyl side chains were introduced at both RH ends, octyl, BO, and HD groups, resulting in **T-2FB-T-ORH**, **T-2FB-T-BORH**, and **T-2FB-T-HDRH**, respectively. The length and size of the alkyl side chains gradually increased from the octyl to the BO and HD groups. The detailed synthetic scheme is shown in Scheme 1. The NMR spectra are provided in Supplementary Materials. The alkyl RH end groups (ORH, BORH, and HDRH) were synthesized from the corresponding alkylamines through a ring-closing reaction [23,35]. Alkylamines of BONH_2_ and HDNH_2_ were synthesized from the corresponding alkyl alcohols through a two-step process. The alkylation of phthalimide was conducted in the presence of PPh_3_ and DIAD [33], followed by hydrolysis using hydrazine monohydrate [34]. **T-2FB-T** was synthesized via a Stille coupling of 1,4-dibromo-2,5-difluorobenzene and 2-(tributylstannyl)thiophene [36]. Then, formylation was performed at both ends of the thiophene moiety in the synthesized **T-2FB-T** unit using 4-formylmorpholine and *n*-BuLi, resulting in the formation of **T-2FBT-T-CHO** [37]. The final products were obtained via the Knoevenagel condensation of **T-2FB-T-CHO** with alkyl RHs [38]. 

The solubility increased in the order **T-2FB-T-ORH** < **T-2FB-T-BORH** < **T-2FB-T-HDRH** as bulky side branches were introduced, which is well matched with the previous report [29]. **T-2FB-T-ORH** had very low solubility, making it difficult to form a uniform film; therefore, further physical property analyses could not be conducted. However, the introduction of the longest and bulkiest HD groups allowed **T-2FB-T-HDRH** to have sufficient solubility in chloroform, enabling film production for physical property analysis and application in OSC devices.

**Figure 1:**
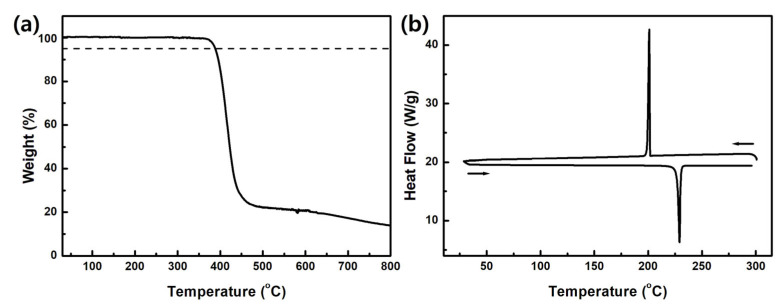
(**a**) TGA and (**b**) DSC curves of **T-2FB-T-HDRH**.

TGA and DSC were performed to determine the thermal characteristics of **T-2FB-T-HDRH**. The results are shown in Figure 1. **T-2FB-T-HDRH** was thermally stable, showing a weight loss (*T*_5d_) of less than 5% up to 388 °C. In DSC, the melting endothermic temperature (*T*_m_) and the exothermic crystallization temperature (*T*_cr_) were measured at 227 and 201 °C, respectively. Despite having long and bulky alkyl side chains, the *T*_m_ of **T-2FB-T-HDRH** was relatively high (*T*_m_ = 204 °C) and is comparable to that of T2-ORH (204 °C), a bithiophene–octylrhodanine-based small molecule [23]. This phenomenon can be attributed to the strong intermolecular interactions of **T-2FB-T-RH** arising from its rod-shaped rigid structure and the introduction of fluorine. 

### 3.2. Optical and Electrochemical Properties 

**Figure 2:**
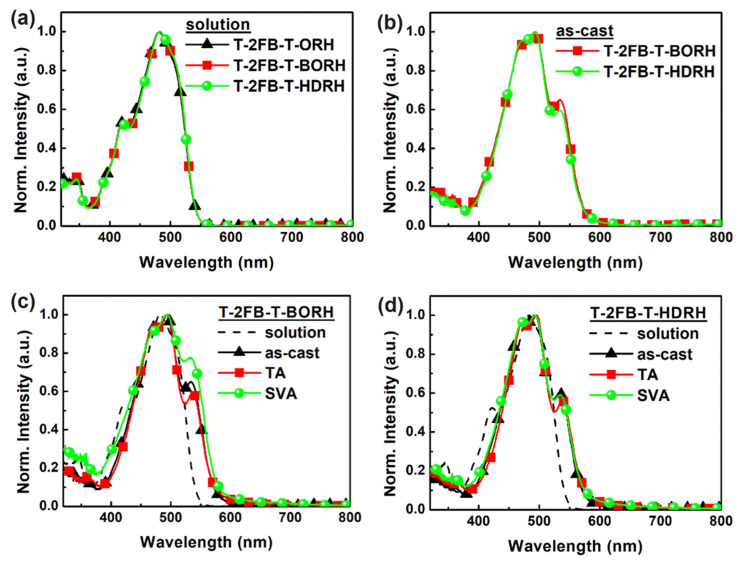
UV−vis absorption spectra of (**a**) the small molecules in the solution, (**b**) the as-cast films of **T-2FB-T-BORH** and **T-2FB-T-HDRH**, and (**c**) **T-2FB-T-T-BORH** and (**d**) **T-2FB-T-HDRH** in solution and film state.

Figure 2 shows the UV–vis absorption spectra of the small-molecule acceptors in the solution and film states. In the chloroform solution (Figure 2a), the three acceptors, consisting of the same molecular backbone, showed almost the same UV–vis spectra, in which their absorption maxima were found at 481 nm (*λ*_max_ = 481 nm). 

The films were prepared by dissolving the solid material in chloroform and spin-coating it onto quartz glass to investigate the optical properties of the film. **T-2FB-T-ORH** could not form appropriate films for UV–vis measurements because of its poor solubility. Figure 2b shows the absorption profiles of the as-cast **T-2FB-T-BORH** and **T-2FB-T-HDRH** films for comparison. As was the case in solution state, the absorption profiles of the two acceptor films were nearly the same, showing an absorption maximum at 494 nm. Compared with the solution state, the as-cast films showed a slight red shift and broad absorption, indicating their *J*-type aggregation. 

Figure 2c,d show the absorption profiles of the **T-2FB-T-BORH** and **T-2FB-T-HDRH** films, respectively. In addition to the as-cast film, the absorption characteristics of the films after SVA or TA treatment, which are the annealing conditions used to fabricate OSC devices, are also shown. As was the case for the solution or as-cast film, the absorption profiles of the two acceptor films appeared similar after annealing. The absorption maxima of the SVA-treated **T-2FB-T-BORH** and **T-2FB-T-HDRH** films were the same at 494 nm. The absorption maxima of the two TA-treated films were also identical at 496 nm. Interestingly, the absorption maxima gradually red-shifted from the as-cast film to the TA film and SVA film, which may be due to the slight increase in molecular aggregation by annealing. Notably, all the acceptor films exhibited complementary absorption to PTB7-Th, the polymer donor used in our OSC fabrication. The optical properties are summarized in Table 1.

### 3.3. Electrochemical Properties 

**Figure 3:**
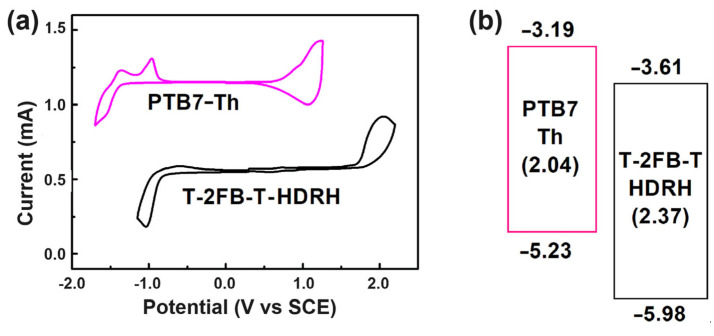
(**a**) CV curve and (**b**) energy level diagram of **T-2FB-T-HDRH**.

The electrochemical properties of the small molecules were characterized by CV measurements and are summarized in Table 2. Figure 3 shows the CV curve of **T-2FB-T-BORH** and ferrocene (as a reference) and the energy level diagrams of **T-2FB-T-BORH** and the polymer donor PTB7-Th. The HOMO and LUMO energy levels of **T-2FB-T-HDRH** were −5.98 and −3.61 eV, respectively. The energy offset between the PTB7-Th polymer donor and **T-2FB-T-HDRH** is sufficient for exciton dissociation in the OSC device. Notably, the LUMO energy level of **T-2FB-T-HDRH** was high-lying (−3.61 eV) compared to those of the other NFAs [6,7], which may be beneficial for device efficiency.

### 3.4. Device Performances

**Figure 4:**
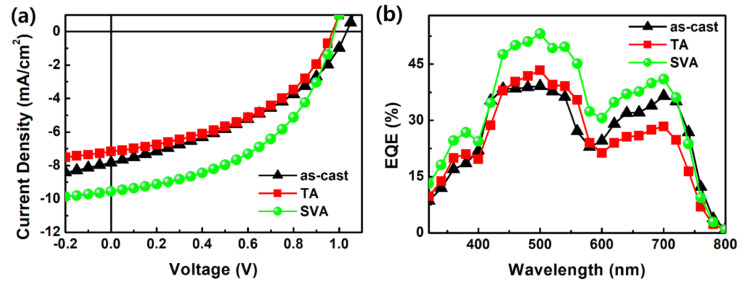
(**a**) *J*–*V* curve and (**b**) *EQE* curve based on **T-2FB-T-HDRH** and PTB7-Th.

The OSC devices were fabricated using an inverted configuration of ITO/ZnO NPs/PEIE/PTB7-Th:T-2FB-T-HDRH (1:2)/MoO*_x_*/Ag. Chloroform was used as the processing solvent, and the devices were subjected to annealing (SVA or TA) treatment to further optimize device performance. Among the three small molecules, only **T-2FB-T-HDRH** was soluble enough to be used for solution-processed device fabrication because of the poor solubility of **T-2FB-T-ORH** and **T-2FB-T-BORH**. The *J*−*V* and EQE curves are depicted in Figure 4, and the photovoltaic properties are summarized in Table 3. 

Devices based on PTB7-Th and **T-2FB-T-HDRH** exhibited photovoltaic properties with PCEs of up to 4.49% under standard AM 1.5G solar irradiation conditions. The PTB7-Th:**T-2FB-T-HDRH** films showed a relatively high open-circuit voltage (*V*_OC_) of over 0.97 V for all the fabrication conditions, which can be explained by the relatively high-lying LUMO energy level of **T-2FB-T-HDRH** (−3.61 eV). Although their efficiency of 4.49% is below the recently reported record OSC performance of over 18%, it is worth noting the substantial variation in material properties and device efficiency resulting from alterations in alkyl chains within a nonfused, simple backbone-based molecule.

**Figure 5:**
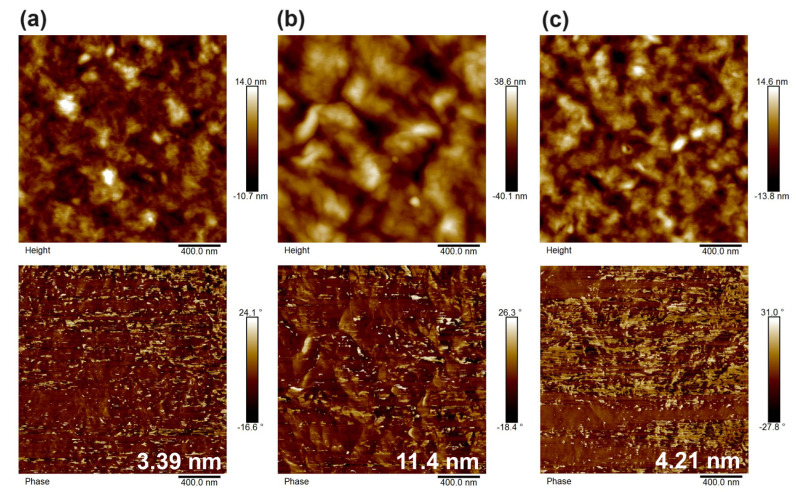
AFM height (top) and phase (bottom) images of the (**a**) as-cast, (**b**) TA-treated, and (**c**) SVA-treated PTB7-Th:**T-2FB-T-HDRH** blend films.

The devices’ efficiency varied depending on the annealing conditions. After SVA treatment, the PCE significantly improved to 4.49% compared to 3.20% for the as-cast film. This was mainly due to the improvement in *J*_SC_ and FF after the SVA treatment. The TA-treated film exhibited a slightly reduced device efficiency (3.12%) compared to that of the as-cast film. The device efficiency, which varies depending on the annealing conditions, can be explained by the differences in the film morphology. As shown in the AFM images (Figure 5), the as-cast and SVA-treated blend films (ITO/ZnO NPs/PEIE/PTB7-Th:**T-2FB-T-HDRH**) exhibited smooth and homogeneous morphologies. The root-mean-square (RMS) roughness of the as-cast and SVA-treated films was 3.39 nm and 4.21 nm, respectively. In contrast, the TA-treated film had a very rough surface, with an RMS roughness of 11.4 nm. As previously mentioned in the DSC results, despite the presence of long and bulky alkyl groups, the rod-shaped structure and introduction of fluorine allowed **T-2FBT-T-HDRH** to have relatively strong intermolecular interactions. In the case of rod-shaped molecules with such strong molecular aggregation, the device performance varies significantly depending on the post-treatment process during device fabrication. A similar phenomenon was observed in our previous study involving a bithiophene-RH-based T2-ORH acceptor (*T*_m_ = 200 °C) [23]. TA treatment caused excessive intermolecular aggregation in the T2-ORH film, resulting in a significantly rough film morphology and decreased *J*_SC_ and thus PCE. However, after SVA treatment, the device efficiency greatly increased owing to the improved charge mobility and appropriate film morphology without excessive aggregation. Similarly, in this study, the charge mobility of **T-2FB-T-HDRH**, which is a rod-shaped acceptor with good crystallinity, was reduced owing to excessive aggregation during TA treatment. However, a smooth and homogeneous film morphology, and thus improved device performance, could be obtained after SVA treatment.

The devices exhibited EQE responses covering 320 and 760 nm, with a peak EQE of 53% centered at approximately 500 nm. The EQE graph is characterized by two peaks centered at approximately 500 and 700 nm, which correspond to the absorption regions of the **T-2FB-T-HDRH** (400–600 nm) and PTB7-Th (600–760 nm) films, respectively. Additionally, after the SVA treatment, the EQE response significantly increased, especially at approximately 500 nm, which corresponded to the contribution of UV–vis absorption by the small-molecule acceptor, which is consistent with the improved film morphology after the SVA treatment. 

In addition, as shown in Table 3, it can be observed that the *V*_OC_ values decreased upon annealing. Such a phenomenon can be attributed to the relationship between *V*_OC_ and diffusion in the junction, as reported in previous studies [39,40]. Additionally, in future work, device engineering, including the integration of ITO-free alternatives like TCOs to enhance device stability concerns [41,42], as well as the incorporation of a broader range of alkyl side chains to further optimize side chain engineering, will be explored.

## 4. Conclusions

Three nonfullerene small-molecule acceptors, **T-2FB-T-ORH**, **T-2FB-T-BORH**, and **T-2FB-T-HDRH**, were synthesized with an T-2FB-T core and alkyl-substituted RH ends. They displayed similar UV–vis absorptions in solution and films, whereas the molecular crystallinity and the materials’ solubility were found to be different depending on the alkyl side chains introduced. The solubility increased in the following order: **T-2FB-T-ORH** < **T-2FB-T-BORH** < **T-2FB-T-HDRH.** This was owing to the introduction of longer and bulkier side chains (octyl < BO < HD). Although **T-2FB-T-ORH** and **T-2FB-T-BORH** could not form uniform films because of their relatively low solubilities, **T-2FB-T-HDRH** had sufficient solubility for device fabrication. OSCs were fabricated with the inverted device configuration ITO/ZnO NPs/PEIE/PTB7-Th:**T-2FB-T-HDRH**/MoO*_x_*/Ag, and the SVA-treated device displayed a PCE of 4.49%, a *V*_OC_ of 0.98 V, a *J*_SC_ of 9.55 mA/cm^2^, and an FF of 48%. After the SVA treatment, the device efficiency increased owing to improved charge mobility and an appropriate film morphology without excessive aggregation, as supported by the AFM study. In this study, the material properties and device characteristics were fine-tuned by modulating the alkyl side chains introduced at the simple thiophene-flanked difluorobenzene backbone. The established correlations between material structure, properties, and device performance in this study could serve as a valuable guideline for future research on organic electronic materials with different backbones.

## Figures and Tables

**Table 1:** Optical properties of the small molecules ^a^.

Acceptor	*λ*_max_ (*λ*_shoulder_) [nm] ^a^				*λ*_onset_ ^b^ [nm]	*E*_g,opt_ ^c^ [eV]
	Solution	As-Cast	SVA	TA		
**T-2FB-T-ORH**	481	-	-	-	-	-
**T-2FB-T-BORH**	482	493 (534)	494 (533)	496 (536)	577	2.15
**T-2FB-T-HDRH**	483	494 (533)	494 (534)	496 (536)	572	2.17

^a^ λ_max_: the absorption maximum; λ_shoulder_: the shoulder peaks; ^b^ *λ*_onset_: the absorption onset wavelength of the as-cast film; ^c^ *E*_g,opt_: the optical bandgap (*E*_g,opt_ = 1240/*λ*_onset_).

**Table 2:** Electrochemical properties of **T-2FB-T-HDRH** ^a^.

Acceptor	*E*_onset,ox_ [V]	*E*_onset,red_ [V]	*E*_HOMO,CV_ [eV]	*E*_LUMO,CV_ [eV]	*E*_g,CV_ [eV]
**T-2FB-T-HDRH**	1.29	–1.08	–5.98	–3.61	2.37

^a^ *E*_onset,ox_ and *E*_onset,red_ [*V* vs. *SCE*]: the onset potential of oxidation and reduction, respectively; *E*_HOMO,CV_ = −(*E*_onset,ox_ − *E*_1/2,ferrocene_ + 4.8) eV, *E*_LUMO,CV_ = −(*E*_onset,red_ − *E*_1/2,ferrocene_ + 4.8) eV, and *E*_g,CV_ = *E*_LUMO,CV_ − *E*_HOMO,CV_.

**Table 3:** Device performance based on PTB7-Th and **T-2FB-T-HDRH**.

	*V*_OC_ [V]	*J*_SC_ [mA/cm^2^]	FF [%]	PCE [%]
As-cast	1.03	7.84	40	3.20
SVA	0.98	9.55	48	4.49
TA	0.97	7.17	45	3.12

## Data Availability

Data are contained within the article and Supplementary Materials.

## Supplementary Materials

The following supporting information can be downloaded at https://www.mdpi.com/article/10.3390/ma17081875/s1, Figure S1. ^1^H NMR spectrum of **HDphth**. Figure S2. ^1^H NMR spectrum of **HDNH2**. Figure S3. ^1^H NMR spectrum of **HDRH**. Figure S4. ^1^H NMR spectrum of **T-2FB-T**. Figure S5. ^1^H NMR spectrum of **T-2FB-T-CHO**. Figure S6. ^1^H NMR spectrum of **T-2FB-BORH**. Figure S7. ^1^H NMR spectrum of **T-2FB-HDRH**.
