# Supplementary material for: Optimizing Alkyl Side Chains in Difluorobenzene–Rhodanine Small-Molecule Acceptors for Organic Solar Cells
Source: Materials (Basel). 2024 Apr 18;17(8):1875. doi: 10.3390/ma17081875 (PMC11052290; doi:10.3390/ma17081875)
Supplement: Supplementary file 1 [file materials-17-01875-s001.zip › materials-2939676-supplementary.pdf]

materials

MDPI

Supplementary Materials

Optimizing Alkyl Side Chains in Difluorobenzene–Rhodanine Small-Molecule Acceptors for Organic Solar Cells

Jongchan Choi [1], Chang Eun Song [2] and Eunhee Lim [3,*]

[1] Department of Chemistry, Kyonggi University, Suwon 16227, Republic of Korea
[2] Korea Research Institute of Chemical Technology, Daejeon 34114, Republic of Korea
[3] Department of Applied Chemistry, University of Seoul, Seoul 02504, Republic of Korea
* Correspondence: ehlim@uos.ac.kr; Tel.: +82-2-2460-2465

1. H NMR spectra

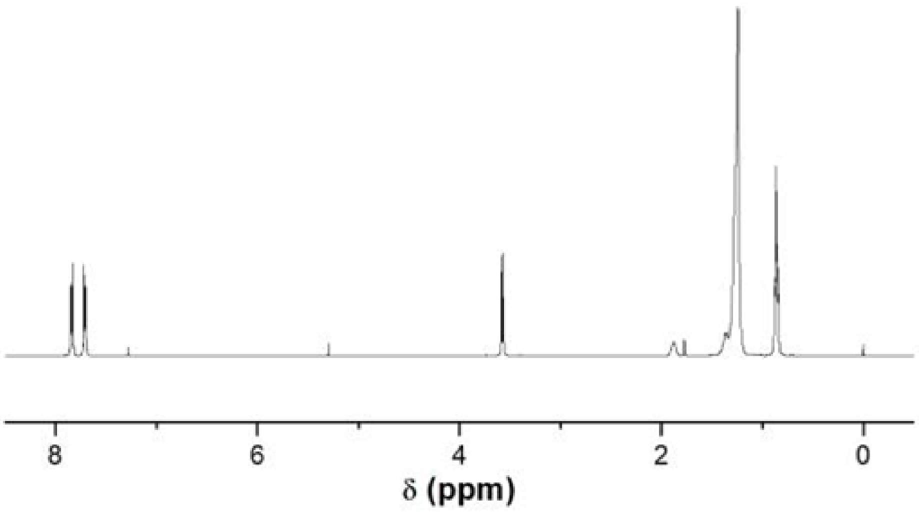

Figure S1. ^{1}H NMR spectrum of **HDphth**.

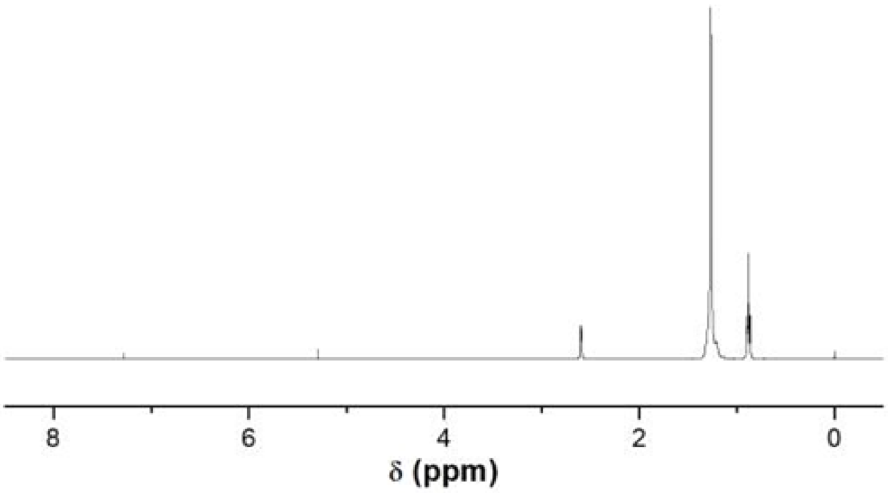

Figure S2. ^{1}H NMR spectrum of **HDNH$_2$**.

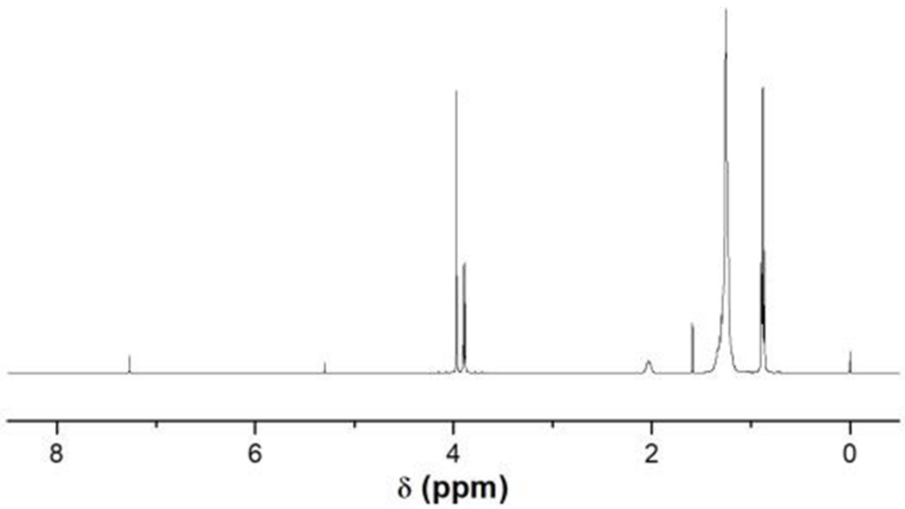

Figure S3. ^{1}H NMR spectrum of **HDRH**.

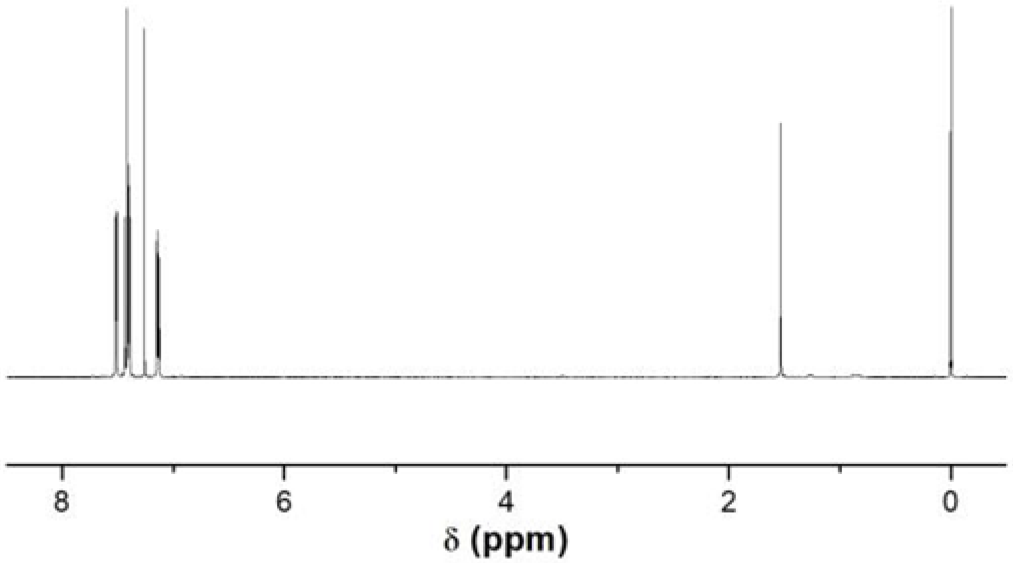

Figure S4. ^{1}H NMR spectrum of **T-2FB-T**.

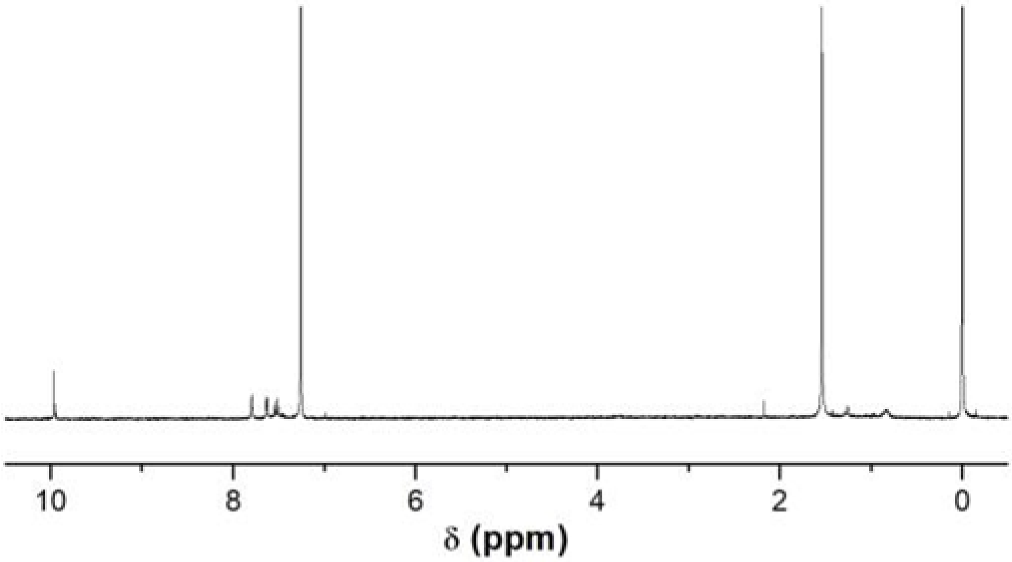

Figure S5. ^{1}H NMR spectrum of **T-2FB-T-CHO**.

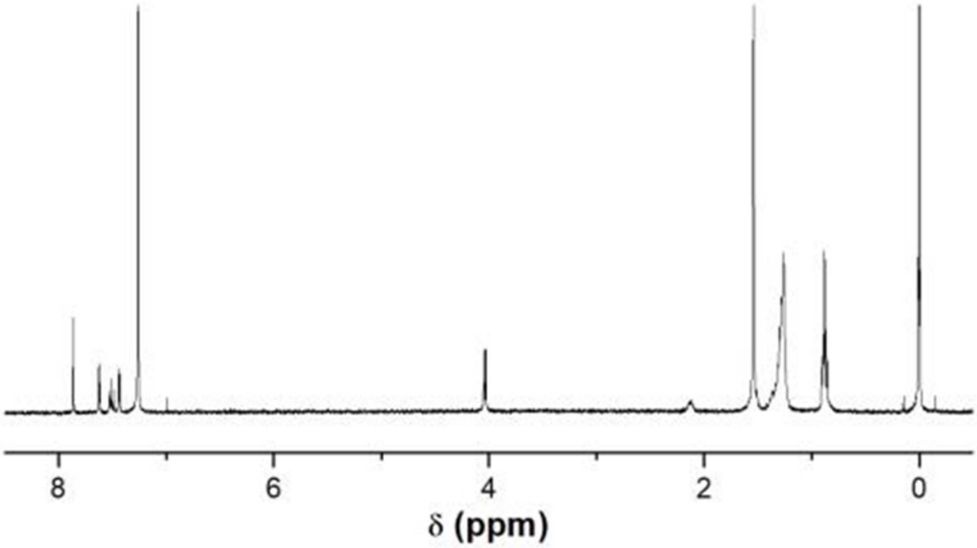

Figure S6. ^{1}H NMR spectrum of **T-2FB-BORH**.

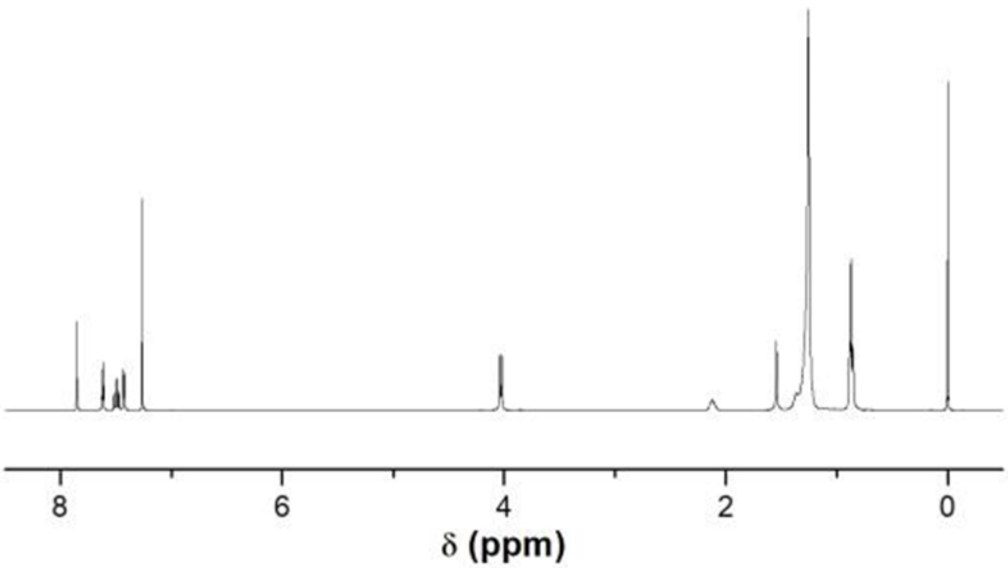

Figure S7. ^{1}H NMR spectrum of **T-2FB-HDRH**.
